# Polytetrafluoroethylene (PTFE) suture vs fiberwire and polypropylene in flexor tendon repair

**DOI:** 10.1007/s00402-021-03899-9

**Published:** 2021-04-19

**Authors:** Elias Polykandriotis, Florian Ruppe, Miriam Niederkorn, Ektor Polykandriotis, Lars Bräuer, Raymund E. Horch, Andreas Arkudas, Jasmin S. Gruener

**Affiliations:** 1 Department of Plastic and Hand Surgery, Friedrich Alexander University Erlangen-Nuremberg FAU, University of Erlangen Medical Center, Krankenhausstr. 12, 91054 Erlangen, Germany; 2 Department of Plastic, Hand and Microsurgery, Sana Hospital Hof, Hof, Germany; 3 Department of Internal Medicine, County Hospital Muenchberg, Muenchberg, Germany; 4 Ross University School of Medicine, Miramar, FL USA; 5 Institute of Anatomy, Chair II; Friedrich Alexander University Erlangen-Nuremberg FAU, Erlangen, Germany

**Keywords:** Flexor tendon repair, Polytetrafluoroethylene (PTFE), Fiberwire, Seramon®

## Abstract

**Background:**

In this study, we evaluate the value of novel suture material based on monofilamentous-extruded polyfluoroethylene (PTFE) compared to polypropylene (PPL) and Fiberwire (FW).

**Materials and methods:**

60 flexor tendons were harvested from fresh cadaveric upper extremities. 4–0 sutures strands were used in the PPL, FW and PTFE group. Knotting properties and mechanical characteristics of the suture materials were evaluated. A 4-strand locked cruciate (Adelaide) or a 6-strand (M-Tang) suture technique was applied as core sutures for a tendon repair. Two-way ANOVA tests were performed with the Bonferroni correction.

**Results:**

Stable knotting was achieved with 5 throws with the PPL material, 7 throws for FW and 9 throws for PTFE. In the PPL group, linear tensile strength was 45.92 ± 12.53 N, in the FW group 80.11 ± 18.34 N and in the PTFE group 76.16 ± 29.10 N. FW and PTFE are significantly stronger than PPL but show no significant difference among each other. Similar results were obtained in the subgroup comparisons for different repair techniques. The Adelaide and the M-Tang knotting technique showed no significant difference.

**Conclusion:**

Fiberwire showed superior handling and knotting properties in comparison to PTFE. However, PTFE allows easier approximation of the stumps. In both, M-Tang and Adelaide repairs, PTFE was equal to FW in terms of repair strength. Both PTFE and FW provide for a robust tendon repair so that early active motion regimens for rehabilitation can be applied.

## Introduction

There is a plethora of evidence stating that early active motion promotes tendon healing and diminishes adhesions after flexor tendon repair [1, 2]. Stress deprivation induces catabolism in tendon cells [3]. The build-up of adhesions may impair range of motion and put additional load on the repair through friction [4]. However, premature and excessive loading through exercise can be detrimental to tendon healing [5].

Fiberwire® (Arthrex Inc, Naples, FL, USA) is a polyblend strand. It is composed of a multi-strand, long-chain ultra-high molecular weight polyethylene (UHMWPE) core with a braided jacket of polyester and UHMWPE. Seramon® is a polytetrafluoroethylene (PTFE) monofilamentous strand produced with a novel production process rendering it substantially stronger than other known PTFE materials (eg. Gore-Tex®). Related substances like Gore-Tex® (expanded polytetrafluoroethylene) or Teflon are widely used in cardiovascular and plastic surgery. There is little foreign body reaction in animal models using the material which stands for an adequate biocompatibility [6–8].

Newer 4–6-strand repairs can achieve an initial tensile strength of up to 100 N (N) [9]. Isolated unresisted flexion of the flexor digitorum profundus can produce peak forces of up to 74 N [10] in vivo. Furthermore, gliding resistance after trauma can grow due to damaged gliding surfaces, posttraumatic edema or a bulging repair [4, 11]. Therefore, the choice of a rehabilitation therapy should correspond to individual patient characteristics, choice of materials used and repair technique. Kannas et al. [12] suggested a rehabilitation approach that includes delayed mobilization for children and adults incapable of following complex regimens. Passive motion is fitting for weaker repairs or other risk factors [13]. Finally, for a 4–6-strand repair, early active motion is the golden standard although results are varied and grossly dependent on rehabilitation [14]. To quote Dr. Peter Amadio: “we did not quite move from no man's land to the promised land” [4].

In non- or minimally displaced bony avulsion of the Flexor digitorum profundus tendon, conservative treatment also can be an option and has a good outcome [15].

Furthermore, if primary reconstruction of the flexor tendon is not possible or led to an insufficient outcome, secondary reconstruction methods can be performed [16]. In additional palmar defects, local flaps like the homodigital neurovascular island flap according to Venkataswami or the neurovascular island flap according to Littler can be an option [17, 18].

## Materials and methods

### Cadaveric flexor tendons

For the purpose of this study, 60 flexor tendons were harvested from non-fixated cadaveric upper extremities. The donor extremities were provided by the Institute of Anatomy, University of Erlangen. The use of the human material was in full compliance with the university policy for use of cadavers and recognizable body parts. For the study, 9 flexor tendons of the fingers and the thumb were utilized as well as the flexor carpi radialis tendon. Six upper extremities from four different donors were used, two female and two male ones. The tendons were obtained from geriatric cadaveric donors with an age range between 65 and 80 years old. Prior to refrigerating, the cadavers were exsanguinated. No deep freezing was performed prior to harvesting of the tendons. The full length of the tendinous part of the units was harvested, to provide for better anchoring onto the measuring device. The tendons were then transected at the middle point by means of a No 11 blade.

### Suturing technique

**Fig. 1:**
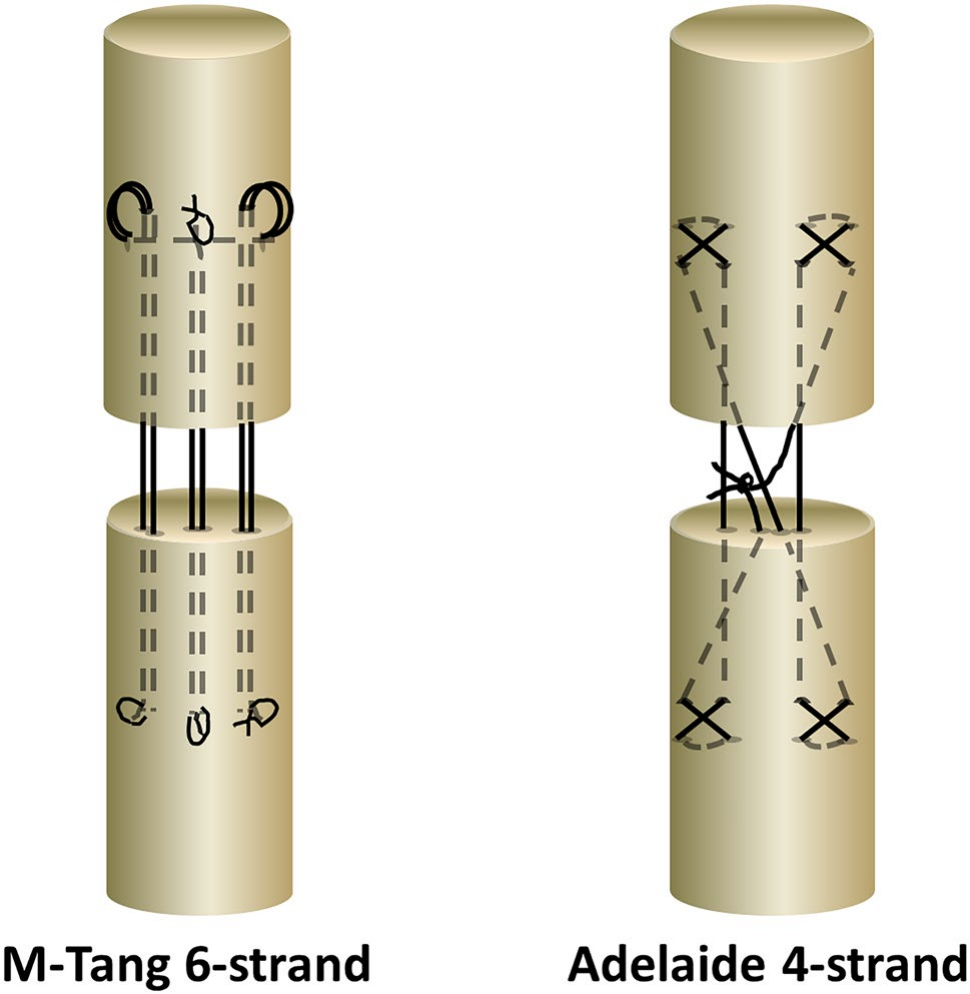
Suture techniques

In literature, many different suture techniques for flexor tendon repair are published and evaluated [19, 20]. In this study, 4-strand locked cruciate (Adelaide) [21] or 6-strand M-Tang [22] suture techniques was applied as core sutures for a single tendon repair as shown in Fig. 1. The repairs were performed on 3 different materials: 4–0 Polypropylene (PPL), 4–0 Fiberwire (FW) and 4–0 Polytetrafluoroethylene (PTFE). The size of every subgroup was *n* = 10. A core suture purchase length of 12 mm was controlled for all groups of tendon repair. The size of locking and grasping anchors was 2 mm. No additional epitendinous suture was performed.

### Measurements of linear tensile strength

For all measurements of linear strength, a universal testing device TIRAtest 28025a, (TIRA GmbH, Schalkau) was used. Testing velocity was set to 300 mm/min. For the measurements, a 1kN modular component was used. The suture material and the repaired tendon were mounted and clamped on both ends. The contact side of each plate had multiple serrations to improve grip on the tendon during testing. The loading continued until failure and the maximum tensile force was noted as ultimate tensile strength.

### Mechanical properties of the suturing materials

Three different strands were used for the tendon repairs: Polypropylene (PPL) (Serapren® USP 4/0, d = 0.185 mm, SERAG-WIESSNER GmbH & Co. KG, Naila, Germany), Fiberwire (FW) (Fiberloop® d = 0.185 mm, Arthrex Inc, Naples, FL, USA) and polytetrafluoroethylene (PTFE), (Seramon® USP 4/0, d = 0.18 mm, SERAG-WIESSNER GmbH & Co. KG, Naila, Germany). In literature, 3–0 or 4–0 strands are recommended [13]. We used 4–0 strands due to bulkiness in the M-Tang technique when using a 3–0 strand. From every charge of the corresponding suture, (*n* = 10) specimens were mounted and measured for linear tensile strength. Subsequently, the suture was divided and knotted with an ascending number of opposing throws. The point was verified when the strand was prone to break at the knot rather than slip (for every group *n* = 3). After finding the minimum of knots needed to prevent slippage, we tested all three 4–0 strands (*n* = 30) for linear strength at the knotting point.

### Statistical analysis

Two-way ANOVA was used for comparison between the groups. Power analysis for a sample size of *n* = 10 for each group, 6 groups altogether, with an estimated effect size of 0.5 and α-value of 0.05 resulted in a power of 0.824. We assumed an estimated delta of 23.5 N between PTFE and PPL due to our previous study [23]. All measurements of tensile strength (failure load) are expressed in Newton (N) with mean values and standard deviation ( ±).

## Results

### Suture materials

**Fig. 2:**
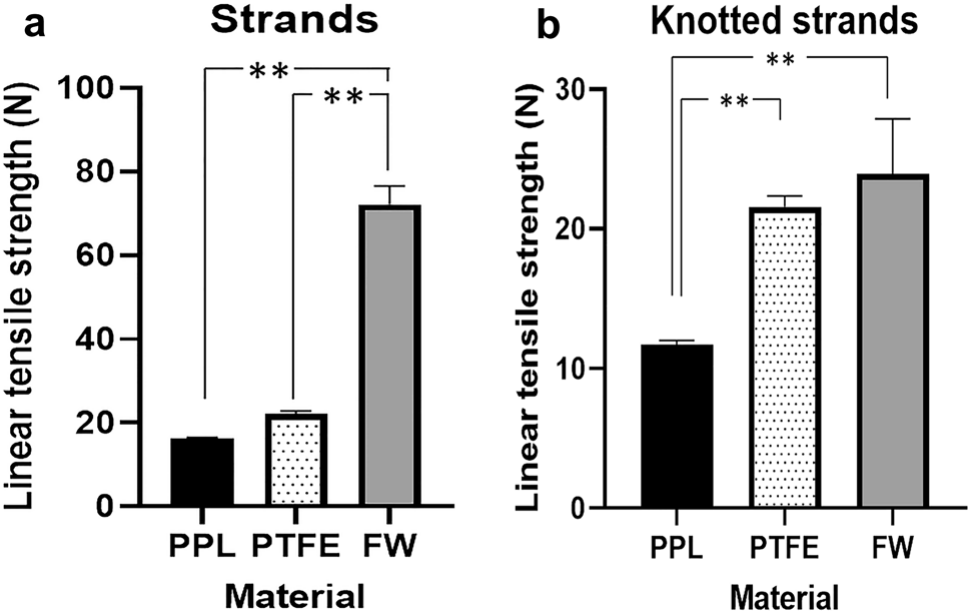
**a**,** b** A single-strand Fiberwire 4–0 (FW) is significantly stronger than the other materials. After knotting, FW loses a part of its linear tensile strength

4–0 PPL strands demonstrated linear tensile strength of 16.37 N ± 0.21. FW demonstrated the highest linear traction strength of 72.16 N ± 4.34. PTFE demonstrated a linear tensile strength of 22.22 N ± 0.69. All comparisons of intergroup linear strength were highly significant (*p* < 0.001). In a preliminary experiment, the number of throws required to achieve a stable knot without the risk of slippage was determined. In PPL, the required number of throws was 5, in FW, it was 7 and in PTFE 9. Knotted PPL displayed a linear strength of 11.71 ± 0.306 N. Knotted FW broke at 23.95 ± 3.920 N and PTFE had a linear tensile strength of 21.57 ± 0.773. The results are summarized in Fig. 2a and b.

### Tendon repairs

Tensile strength of PPL 4–0 suture with the Adelaide repair was 39.69 N (30.88–47.74 ± 5.57). Tensile strength of PPL 4–0 suture with the M-Tang repair was 52.14 N (33.44–76.42 ± 14.21).

Tensile strength of Fiberwire 4–0 suture with the Adelaide repair was 70.96 N (51.51–111.47 ± 21.18). Tensile strength of Fiberwire 4–0 suture with the M-Tang repair was 89.25 N (76.87–100.61 ± 8.68).

Tensile strength of PTFE 4–0 suture with the Adelaide repair was 72.79 N (14.37–100.29 ± 27.91). Tensile strength of PTFE 4–0 suture with the M-Tang repair was 80.97 N (42.810– 137.75 ± 30.55). There was no significant difference between Adelaide and M-Tang repair within any of the material groups. However, both FW and PTFE proved to be significantly stronger than PPL in both settings. No significant difference could be detected among FW and PTFE. A summary of the results is demonstrated in Table 1.

**Table 1:** Summary of results from flexor tendon repairs

	Polypropylene (PPL)	Fiberwire (FW)	Polytetrafluoro-ethylene (PTFE)	*p*
M-Tang 6-strand	52,14 ± 14.21 N	89.25 ± 8.68 N	80.97 ± 30.94 N	PPL-FW < 0.001** PPL-PTFE 0.0079** FW-PTFE > 0.99
Adelaide 4-strand	39.69 ± 6.57 N	70.96 ± 21.18 N	72.79 ± 27.91 N	PPL-FW 0.0036** PPL-PTFE 0.0019** FW-PTFE > 0.99
*p*	0.53	0.15	> 0.99	
Pooled data Adelaide + M-Tang	45.92 ± 12.53 N	80.11 ± 18.34	76.16 ± 29.10	PPL-FW < 0.001** PPL-PTFE < 0.001** FW-PTFE > 0.99
Linear tensile strength of solitary strand	16.37 N ± 0.21	72.16 N ± 4.34	22.22 N ± 0.69	All comparisons < 0.001**

**Fig. 3:**
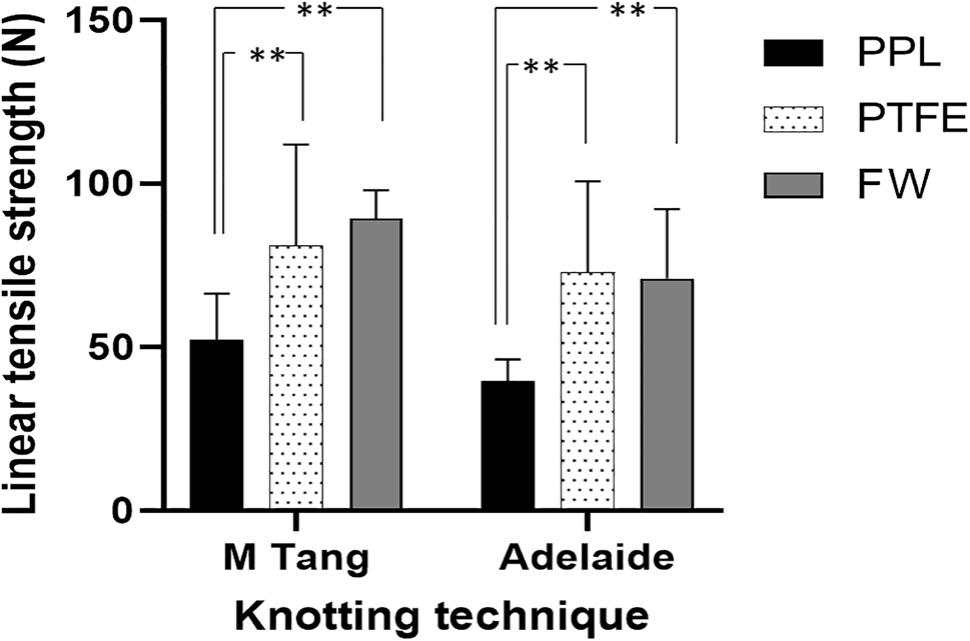
Tensile strength of flexor tendon repairs with two different multi-strand core sutures techniques. The error indicator depicts Standard deviation. **Highly significant. *PPL *Polypropylene, *PTFE *Polytetrafluoroethylene, *FW *Fiberwire

Repairs with PTFE displayed a peak tensile strength comparable to FW. Both repairs were significantly stronger than those with PPL Fig. 3.

Both, knot breakage and pullout occurred. Repair failure due to pullout was 10% in the polypropylene group, 30% in the PTFE group and 50% in the Fiberwire group.

## Discussion

In a previous study [23], we tested PTFE against PPL using a standard 2-strand flexor repair technique. All tendon repairs performed with PTFE in the previous study had failed due to cheese wiring. The limiting point of the material itself was the sleekness leading to problems in handling. In this study, we further investigate PTFE as an option for a flexor tendon repair, performing stronger and more advanced repair techniques. Furthermore, a comparison against FW seemed meaningful since its use is gaining popularity [9, 13].

Testing of the solitary strands with or without prior knotting provided important insights. With constant linear tensile strength and without knotting, FW proved to be significantly stronger than the two other materials. However, after knotting this difference vanishes, indicating that FW is prone to mechanical distortion. Knotting to some extend inflicts distortion of a strand and therefore the knot itself presents a breaking point. This effect seems to be profound in the FW material, nevertheless, even after knotting FW retains excellent tear strength properties. On the contrary, there was no loss of tensile strength by knotting for the PTFE strand. A degree of plastic distortion is necessary for a knot to hold, so it is no surprise that 9 or more throws of a knot are needed for PTFE to hold. In this experimental setting, we expected tensile force measurements of more than 100 N. Knotting with 9 throws was necessary for this experimental setting. We expect that a flexor tendon repair with 5–6 knots will be adequate in the clinical setting. However, even 5–6 knots are quite bulky.

We first faced difficulty handling the PTFE material due to very low surface friction and a “slippery” feeling. Strict training was required to handle the comparatively slick material. On the other hand, approximating the tendon stumps and tightening of the repair went smoother, affording less tissue trauma. The material was very pliable in great contrast to the ominously rigid PPL but also in comparison to FW. With the M-Tang technique combined with FW, there was some difficulty adjusting the tension of the last 2 strands to the tension of the initial 4 strands. Finally, the knot of the PTFE strand ended up being very bulky in comparison to the other materials, however, with the Adelaide technique, the knot can be buried between the stumps.

The results indicate that when FW or PTFE strands are used, the initial repair strength is adequate for early active motion rehabilitation. As previously shown, active finger flexion exposes uninjured flexor tendons to forces of up to 20 N [10, 24]. Conversely, isolated flexion of the deep flexors, generates increased loads to a maximum of 75 N. Additionally, subsequent to an injury, tendon load rises due to adhesions and higher gliding resistance [4]. Amadio et al. further assumed a 30% loss of strength due to gapping and another loss of 10–20% secondary to softening of the tendon. Amadio also coined the terms “low friction repair” and “safe zone” defining the range where the load placed on a tendon will set it in motion but will not evoke gapping or rupture. The ideal repair is strong enough and low on friction so as to “expand” this safe zone and allow for a safe early active motion rehabilitation regimen [12].

The PTFE material (Seramon®) in our study displayed an array of positive properties [25]. It is distinct to other commercial PTFE strands, e.g. Gore-Tex®. Under laboratory conditions, Seramon® proved to be significantly superior to Gore-Tex® in terms of linear tensile strength and knotted strand strength owing to a different manufacturing process. This material despite being more pliable than PPL or FW shows minimal distortion after knotting and minimal elongation upon linear traction [23]. As a dual benefit, the knot is less of a breaking point and the risk of gapping is minimized [26]. In addition, PTFE is biologically inert [27] and less likely to cause inflammation [5, 28]. Finally, as a monofilamentous material, it is less prone to infection [29].

It has to be underlined that also the flexor carpi radialis tendon was used which could lead to discrepancies and adds an additional variable. Also, the flexor tendons of the thumb were used, which can additionally cause a discrepancy. Yet, the different tendons were distributed evenly in the groups. However, regarding the high number of trials, significant results and, respectively, low standard deviation, there seem to be no major differences between the used types of flexor tendons.

A certain limitation of the study has to be mentioned as well: this study did not assess the capacity of gap resistance of flexor tendon repair, such as gap formation force and stiffness of tendon repair.

## Conclusion

Mechanically is PTFE equal to FW, providing for a robust flexor repair capable of supporting early active motion. However extremely low surface friction properties render multiple bulky knotting necessary. Adaptation of surgical technique would be required. However, approximating the tendon stumps and tightening of the tendon repair went smooth which should cause less tissue trauma.

In summary, we hold PTFE strands suitable for a clinical trial on flexor tendon repair.
